# Vestibular Patient Journey: Insights From Vestibular Disorders Association (VeDA) Registry

**DOI:** 10.1002/acn3.70334

**Published:** 2026-02-18

**Authors:** Ali Rafati, Sami I. Nassar, Shaun A. Nguyen, Patricia L. Gerend, Michael C. Schubert, Michael T. Teixido, Frederick A. Godley, Joel A. Goebel, Cynthia A. Ryan, Habib G. Rizk, Amir Kheradmand

**Affiliations:** ^1^ Department of Neurology Johns Hopkins University School of Medicine Baltimore Maryland USA; ^2^ Department of Otolaryngology – Head and Neck Surgery Medical University of South Carolina Charleston South Carolina USA; ^3^ Vestibular Disorders Association (VeDA) Portland Oregon USA; ^4^ Department of Otolaryngology – Head and Neck Surgery Johns Hopkins University School of Medicine Baltimore Maryland USA; ^5^ Department of Otolaryngology University of Pennsylvania Philadelphia Pennsylvania USA; ^6^ Association of Migraine Disorders North Kingstown Rhode Island USA; ^7^ University Otolaryngology Providence Rhode Island USA; ^8^ Department of Otolaryngology‐Head and Neck Surgery Washington University School of Medicine St. Louis Missouri USA; ^9^ Department of Neuroscience Johns Hopkins University School of Medicine Baltimore Maryland USA

**Keywords:** dizziness, imbalance, vertigo, Vestibular disorders

## Abstract

**Objective:**

Vestibular symptoms impose a high burden of disability. Understanding real‐world diagnostic and treatment pathways can identify care gaps and guide interventions. We aimed to characterize symptom profiles, diagnostic trends, provider involvement, and treatment patterns in vestibular disorders. We also examined whether these real‐world pathways align with expected clinical features in vestibular diagnoses.

**Methods:**

Data from the Vestibular Disorders Association (VeDA) patient registry from March 21, 2023, to January 29, 2024 was analyzed, focusing on vestibular diagnoses, symptom patterns, care pathways, treatments, and diagnostic predictors.

**Results:**

Of 172 respondents (75.6% female), 66.3% reported multiple vestibular diagnoses (mean 1.8) and 18.9% reported inability to work. The most prevalent diagnoses were vestibular migraine (VM, 50.6%), benign paroxysmal positional vertigo (BPPV, 36.0%), Meniere's disease (26.9%), persistent postural‐perceptual dizziness (PPPD, 20.9%), and vestibular neuritis (VN, 21.6%). VM overlapped with BPPV (44.8%), PPPD (31.0%), and Meniere's disease (27.6%). Patients consulted an average of ~15 providers, underscoring the combined effects of participation bias, diagnostic overlap, and fragmented care pathways characteristic of vestibular conditions. Symptoms, triggers, functional impact, provider involvement, and treatments were broadly similar across diagnoses. Logistic regressions revealed that VM was associated with frequent headaches, spontaneous vertigo, and frequent nausea/vomiting (ORs 24.70, 21.07, 5.58; *p* = 0.003, 0.02, 0.036), Meniere's with dietary improvement (OR 38.00; *p* = 0.016), PPPD with VM overlap and visual‐induced vertigo (ORs 6.83, 0.02; *p* = 0.03, 0.02), and VN with the lowest frequency of autonomic symptoms (OR 0.00006; *p* = 0.005).

**Interpretation:**

The findings reveal a complex clinical landscape of vestibular disorders marked by multiple diagnoses and barriers to effective care. Overlapping symptom profiles across disorders further complicate diagnostic accuracy. Although regression analysis confirmed diagnosis‐specific features, neither provider involvement nor treatments aligned consistently with diagnoses, highlighting fragmented care pathways. These findings call for clearer diagnostic pathways, enhanced provider training, and coordinated multidisciplinary care to improve diagnostic accuracy and treatment for vestibular disorders.

## 1 Introduction

Vestibular symptoms are a common yet complex clinical challenge across healthcare settings. They affect up to 7.4% of adults over a lifetime, with annual prevalence and incidence rates of 5% and 1.4%, respectively [1]. Often presenting as vertigo, dizziness, or imbalance, these symptoms are typically more than a fleeting discomfort. They reflect disruption in complex neural processes that enable coherent spatial perception and interaction with the surrounding environment. These symptoms can severely affect quality of life and are frequently linked to elevated anxiety and depression [2, 3]. In this context, the burden of vestibular dysfunctions extends beyond physical discomfort, affecting both emotional well‐being and functional independence.

Despite their high prevalence, vestibular disorders are often underdiagnosed or misdiagnosed. This is largely due to the heterogeneous and non‐specific nature of these symptoms, making both diagnosis and treatment challenging. For example, while the term “dizziness” is used broadly, it may hold different meanings for different individuals. For some, it may indicate a problem with the vestibular system, but others may use it to describe sensations like lightheadedness, unsteadiness, detachment, confusion, or other nonspecific discomforts. These vague descriptions offer limited diagnostic value compared to more specific features like symptom triggers, duration, or exacerbating factors [4]. Such ambiguity can be frustrating for both patients and clinicians, contributing to multiple consultations, repeated diagnostic testing, and delays in appropriate treatments [5, 6, 7].

A particularly underexplored challenge in vestibular care is the high prevalence of multiple, and sometimes conflicting, diagnoses in individual patients [6, 7]. This pattern reflects not only the inherent ambiguity of vestibular symptoms but also the variability in diagnostic approaches across different specialties. The frequency and clinical context of these overlapping diagnoses can offer valuable insight into the fragmentation of care and the diagnostic uncertainty commonly experienced by patients with vestibular symptoms. To this end, patient‐reported data can capture shared experiences and reveal the real‐world complexity of vestibular disorders within the healthcare system.

Patients with vestibular symptoms often struggle to carry out daily activities and meet work‐related responsibilities, frequently resulting in medical leave and increased financial strain [8, 9]. These challenges are often compounded by higher use of medical resources, including repeated clinical visits, diagnostic tests, hospitalizations, and trial‐and‐error treatment approaches [7, 10, 11, 12]. For individuals with limited financial resources or inadequate insurance coverage, these burdens can significantly delay or even prevent access to appropriate care [13]. While these trends have been increasingly recognized, detailed insights into the specific impact and clinical characteristics of individual vestibular disorders remain limited. The present study aims to address this gap by drawing from the Vestibular Disorders Association (VeDA) patient registry, which captures the experiences and perspectives directly reported by patients. The registry collects a broad array of patient‐reported information, including symptom profiles, healthcare encounters, diagnoses, treatments, barriers to care, and quality of life indicators.

To better understand the complexities of medical care for vestibular patients, here we examined the pathways to diagnosis and treatment using detailed patient‐reported data. We analyzed symptom profiles—including their quality, timing, duration, and triggers—and investigated how initial symptom presentations corresponded to common vestibular diagnoses. We also assessed the types of treatments patients reported and whether they were effective. In addition, we analyzed provider specialties involved in diagnosis and treatment to examine potential influences on clinical outcomes. To provide a more comprehensive view of patient experiences, we also assessed barriers to care and quality of life measures, capturing the psychosocial challenges associated with their symptoms. Collectively, these insights can provide an in‐depth view of the patient journey—from symptom onset to diagnosis and treatment—and highlight critical opportunities for improving diagnostic accuracy, care coordination, and patient‐centered outcomes.

## 2 Methods

### 2.1 Study Design and Data Collection

**FIGURE 1:**
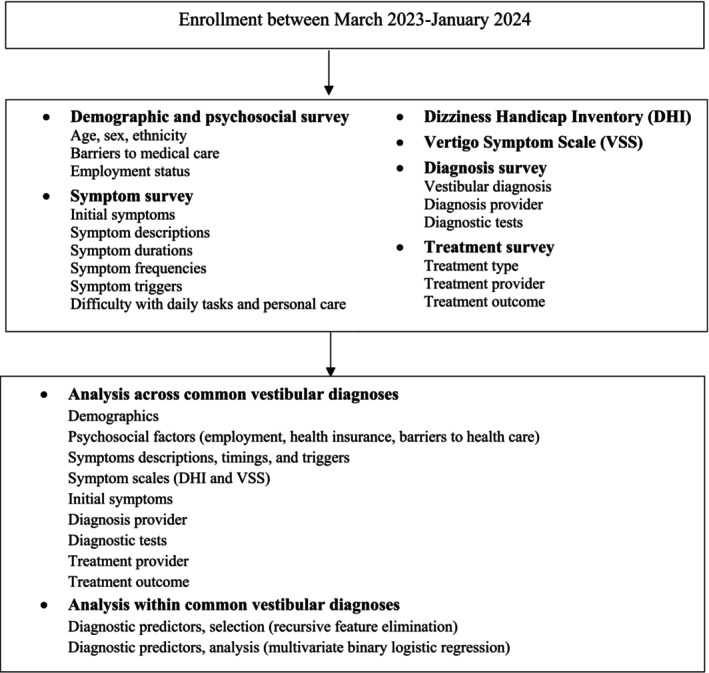
Study Flow diagram showing the structure of the VeDA registry and data analysis.

This is a cross‐sectional study from the VeDA patient registry aimed at understanding the diagnostic and treatment journey of individuals with vestibular disorders. Respondents were recruited through the VeDA website, social media, and referrals from healthcare providers. The study protocol was approved by the Genetic Alliance Institutional Review Board. Eligibility criteria included adults with self‐reported vestibular symptoms and diagnoses. Figure 1 shows a schematic illustration of the VeDA registry and patient enrollment for this study. Between March 21, 2023, and January 29, 2024, adults experiencing vestibular symptoms completed online questionnaires through the Luna registry platform, part of the Genetic Alliance Platform for Engaging Everyone Responsibly (PEER) network.

The registry collected a comprehensive range of data including demographics, symptom characteristics (description, timing, duration, and triggers), diagnostic tests, diagnoses, treatments, provider specialties, barriers to care, medical and family history, quality of life metrics, and validated symptom scales including the Vertigo Symptom Scale (VSS) [14], and the Dizziness Handicap Inventory (DHI) [15] (Figure 1). The VSS is a self‐report questionnaire investigating symptoms of balance disorder, somatic anxiety, and autonomic arousal in patients complaining of dizziness and vertigo [14]. The VSS is scored from 0 to 60, with higher scores indicating greater severity [14], and scores of ≥ 12 represent severe symptoms [16]. The DHI quantifies the impact of vestibular symptoms on daily life by measuring self‐perceived handicaps. DHI is interpreted as mildly handicapped with scores of 16–34, moderately handicapped with scores of 36–52, and as severely handicapped with scores > 54 [15]. Patients reported their symptom characteristics using definitions provided by the Bárány Society's international classification [17]. Dizziness was defined for respondents as a disturbed sense of spatial orientation without any perception of motion. Vertigo was defined as a false sensation of movement of self or the environment, and lightheadedness as a sensation of near‐fainting. Autonomic symptoms were defined as sweating, fainting or blacking out, heart pounding or fluttering, and hot or cold spells.

### 2.2 Feature Selection and Statistical Analysis

Continuous variables were reported using mean and standard deviation (SD), and categorical variables were reported using counts and percentages. Normality was checked using the Kolmogorov‐Smirnov test. Continuous variables were compared using one‐way ANOVA, and categorical variables were compared using the Chi‐squared test. The Bonferroni post hoc test was used for pairwise comparisons.

To evaluate the predictive value of the large number of variables within the dataset, the Recursive Feature Elimination (RFE) was applied to isolate the most informative predictors for the top five diagnostic outcomes. As a wrapper‐based feature selection method, RFE iteratively removes the least important variables based on model performance until the optimal subset of features is selected [18]. To ensure model robustness and limit overfitting, RFE was applied with 10‐fold cross‐validation. At each step, the model was retrained on the remaining features, and its predictive performance was evaluated by computing the mean cross‐validated classification accuracy and standard deviation. For each diagnostic outcome, the subset of clinical predictors that achieved the highest mean cross‐validated accuracy was selected as the optimal set for further analysis.

To determine the appropriate number of variables within each diagnostic outcome, we then applied an events‐per‐variable (EPV) criterion, which considers the number of cases (events) per outcome. With this approach, an EPV between 5 and 10 is generally considered acceptable for predictive modeling [19, 20]. This analysis was performed for the five most common diagnoses. The EPV value calculated for each diagnosis was used to cap the number of features yielded from the RFE. The final set of variables yielded from the RFE was then entered into a multivariate binary logistic regression model for each diagnosis. Adjusted odds ratios and 95% confidence intervals were derived to provide interpretable measures of association and clinical inference. The “caret” package was used for model validation and RFE execution. All analyses were performed using RStudio (v4.5.0; RStudio Team, 2025).

## 3 Results

### 3.1 Demographics and General Characteristics

Of 272 patients who initially registered, 172 respondents reported their diagnoses and completed all questionnaires. Most participants were female (75.6%), white (84.3%), non‐Hispanic (94.5%), and between 45 and 64 years of age (50.4%). On average, patients reported consulting with 15 providers (SD = 11) for their symptoms. Overall, 66.3% reported having multiple diagnoses (Table S1). The average number of diagnoses was 1.80 (SD = 1.41) per individual patient. Otolaryngologists (i.e., general otolaryngologists, otologists, or neurotologists) were the most frequently reported diagnosing providers, accounting for 52.3% of patients, followed by neurologists at 16.9% and primary care providers (PCP) at 5.8%. Regarding the frequency of medical visits, 48.4% reported monthly or less frequently, 32.2% yearly or less, 11.8% monthly, 3.2% weekly or less, and 7.6% reported no continuity of care at all (Table S1). Inability to work was reported by 18.9% of patients. Retirement was the most common employment status at 35.4%, while student status was the least common at 2.4% (Table S1).

### 3.2 Diagnosis, Prevalence, and Symptom Profiles

The most prevalent diagnosis was vestibular migraine (VM) at 50.6%, followed by benign paroxysmal positional vertigo (BPPV) at 36%, Meniere's disease at 26.9%, persistent postural‐perceptual dizziness (PPPD) at 20.9%, and vestibular neuritis (VN) at 21.6% (Table S1). Table 1 compares demographic data, and Table 2 summarizes clinical and symptom profiles across these five common vestibular diagnoses. There was no significant difference in sex (*p* = 0.09) or age (*p* = 0.83) distribution (Table 1). The most common comorbidity was between VM and BPPV, with 44.8% of VM patients also diagnosed with BPPV. This was followed by comorbidity of VM with PPPD at 31.0% and with Meniere's disease at 27.6% (Table S2). Because diagnoses are self‐reported, the analysis did not capture which specialties confirmed multiple diagnoses or whether standardized diagnostic criteria were consistently applied.

**TABLE 1:** Demographics, employment status, and barriers to medical care among the top five prevalent diagnoses.

Variables	VM	BPPV	Meniere's disease	PPPD	VN	*p* ^a^
Age groups						0.834
18–24 years	1.7	0	0	0	0	
25–44 years	18.6	12.5	13.3	13.6	13.0	
45–64 years	55.9	52.5	56.7	59.1	39.1	
> 64 years	23.7	35.0	30.0	27.3	47.8	
Sex (female)	83.1	82.5	60.0	86.4	78.3	0.090
Employment	57.6	47.5	46.7	50	43.7	0.890
Unable to work (disabled)	16.9	17.5	20.0	13.6	4.3	
Retired	23.7	35.0	33.3	36.4	39.1	
Self‐employed	15.3	17.5	6.7	18.2	17.4	
Employed part‐time or full‐time	39.0	27.5	40.0	31.8	30.4	
Out of work for 1 year or more	1.7	0	0	0	4.3	
Student	3.4	2.5	0	0	4.3	
Barriers to medical care						0.269
Long waitlist to see a provider or couldn't find a qualified provider in the area	14.1	8.8	15.6	17.6	19.4	
Told by provider that symptoms would go away on their own or was misdiagnosed	21.2	12.3	11.1	11.8	16.7	
Believed symptoms would go away on their own or simply needed to change lifestyle	11.8	8.8	6.7	20.6	19.4	
Didn't know enough about what caused symptoms to advocate for self, or didn't know which type of provider to see, or how to find them.	30.6	31.6	24.4	35.3	2.8	
Did not seek care in a timely manner or could not take time off from work	3.5	3.5	0	0	19.4	
No health insurance	0	1.8	2.2	2.9	0	
None	18.8	33.3	40.0	11.8	22.2	

Abbreviations: BPPV, benign paroxysmal positional vertigo; PPPD, persistent postural perceptual dizziness; VM, vestibular migraine; VN, vestibular neuritis. All values are presented as percentages.

^a^ *p*‐value for the Chi‐squared test.

**TABLE 2:** Symptom characteristics among the top five prevalent diagnoses.

Variables	VM	BPPV	Meniere's disease	PPPD	VN	*p* ^a^
Description of vestibular symptoms						0.281
Vertigo (a false sensation of movement of self or the environment)	14.9	14.5	19.6	13.9	21.6	
Lightheadedness (a sensation of near fainting)	24.1	40.3	23.9	27.8	32.4	
Imbalance or unsteadiness	13.8	6.5	4.3	16.7	13.5	
Dizziness (a sense of spatial disorientation)	10.3	6.5	6.5	8.3	8.1	
A tilt of the visual environment	6.9	8.1	8.7	2.8	8.1	
A sense of body pulling or tilting	2.3	6.5	2.2	5.6	13.5	
A sense of movement of the visual field or jumping in vision (oscillopsia)	13.8	8.1	21.7	16.7	0	
A lag or blurriness of vision with head movement	12.6	9.7	10.9	8.3	0	
None	1.1	0	2.2	0	2.7	
Description of imbalance						0.605
Sense of unsteadiness	40.7	41.9	38.6	38.9	40.0	
Sense of being pushed or pulled	22.1	11.3	6.8	27.8	20.0	
Near fall	18.6	19.4	27.3	13.9	14.3	
Fall	15.1	19.4	22.7	16.7	22.9	
No imbalance	3.5	8.1	4.5	2.8	2.9	
Dizziness triggers						0.353
Spontaneous without any trigger	19.5	19.6	37.2	8.6	11.1	
Sound	8.0	7.1	7.0	5.7	8.3	
Straining	4.6	7.1	4.7	5.7	0	
Visual stimulation	21.8	17.9	11.6	28.6	22.2	
Positional changes	27.6	23.2	27.9	31.4	25.0	
Head movement	18.4	25.0	11.6	20.0	33.3	
Vertigo triggers						0.391
Spontaneous without any trigger	25.6	16.7	34.1	10.3	10.0	
Sound	6.1	5.6	7.3	3.4	16.7	
Straining	2.4	3.7	0	6.9	3.3	
Visual stimulation	19.5	20.4	14.6	10.3	10.0	
Positional changes	31.7	35.2	29.3	48.3	40.0	
Head movement	14.6	18.5	14.6	20.7	20.0	
Frequency of vestibular symptoms						0.263
Continuous	39.1	35.5	39.1	52.8	37.8	
Episodic	59.8	59.7	50.0	41.7	54.1	
Resolved	1.1	4.8	10.9	5.6	8.1	
Duration of vestibular symptoms						0.291
Never	0	3.2	0	2.8	0	
<5 min	10.3	12.9	4.3	8.3	13.5	
5–20 min	6.9	1.6	2.2	8.3	5.4	
21 min–72 h	54.0	46.8	60.9	33.3	40.5	
Continuous	28.7	35.5	32.6	47.2	40.5	
Inability to stand						0.624
Never	9.2	14.5	8.7	16.7	16.2	
Occasional	65.5	62.9	67.4	47.2	51.4	
Often (weekly)	12.6	11.3	17.4	16.7	21.6	
Frequent (daily)	12.6	11.3	6.5	19.4	10.8	
Autonomic symptoms						0.225
Never	5.7	11.3	6.5	11.1	21.6	
Occasional	46.0	48.4	45.7	58.3	48.6	
Often (weekly)	28.7	27.4	30.4	22.2	27.0	
Frequent (daily)	19.5	12.9	17.4	8.3	2.7	
Nausea or vomiting						0.136
Never	10.3	22.6	4.3	13.9	24.3	
Occasional	57.5	54.8	67.4	44.4	54.1	
Often (weekly)	20.7	14.5	21.7	22.2	13.5	
Frequent (daily)	11.5	8.1	6.5	19.4	8.1	
Headache						0.110
Never	4.6	12.9	4.3	11.1	18.9	
Occasional	37.9	43.5	47.8	30.6	43.2	
Often (weekly)	28.7	19.4	28.3	16.7	21.6	
Frequent (daily)	28.7	24.2	19.6	41.7	16.2	
Photophobia						0.239
Never	8.0	22.6	15.2	11.1	21.6	
Occasional	31.0	32.3	34.8	33.3	45.9	
Often (weekly)	35.6	22.6	30.4	25.0	18.9	
Frequent (daily)	25.3	22.6	19.6	30.6	13.5	
Phonophobia						0.774
Never	9.2	19.4	13.0	19.4	21.6	
Occasional	34.5	35.5	30.4	27.8	37.8	
Often (weekly)	31.0	22.6	30.4	22.2	21.6	
Frequent (daily)	25.3	22.6	26.1	30.6	18.9	
Difficulty with daily tasks or personal care						0.974
Never	3.8	3.9	5.0	3.1	2.9	
Occasional	62.0	68.6	65.0	65.6	68.6	
Often (weekly)	21.5	9.8	15.0	15.6	11.4	
Frequent (daily)	12.7	17.6	15.0	15.6	17.1	
Symptoms scales						
Mean DHI score (SEM)	47.76 (2.21)	44.82 (2.70)	44.76 (3.23)	50.21 (3.53)	44.69 (3.12)	0.652^b^
Mean VSS score (SEM)	21.73 (1.17)	17.76 (1.46)	20.50 (1.59)	20.79 (2.14)	17.56 (2.17)	0.199^b^

Abbreviations: BPPV, benign paroxysmal positional vertigo; DHI, Dizziness Handicap Inventory; PPPD, persistent postural perceptual dizziness; SEM, standard error of mean; VM, vestibular migraine; VN, vestibular neuritis; VSS, Vertigo Symptom Scale. All values are presented as percentages unless otherwise stated.

^a^ *p*‐value for the Chi‐squared test unless stated otherwise.

^b^ *p*‐value for ANOVA test.

Overall, there were no significant differences across the diagnoses in how patients described their vestibular symptoms (*p* = 0.281) and imbalance (*p* = 0.605). Notably, lightheadedness was described at 40.3% in BPPV, 32.4% in VN, 27.8% in PPPD, 24.1% in VM, and 23.9% in Meniere's, followed by unsteadiness at 16.7% in PPPD and 13.8% in VM, visual tilt at 8.7% in Meniere's, and body tilting or pulling at 13.5% in VN (Table 2). Imbalance was also mainly described as a sense of unsteadiness across all the diagnoses (~40% of patients), followed by a sense of being pushed or pulled, with 22.1% in VM, 27.8% in PPPD, and 20% in VN. Near falls were reported in Meniere's at the rate of 27.3%, while actual falls were at the rate of 22.9% in VN, followed by 22.7% in Meniere's (Table 2).

Symptom triggers were similarly distributed across diagnostic groups, with no significant difference in dizziness (*p* = 0.353) or vertigo (*p* = 0.391). Meniere's had a relatively high rate of spontaneous dizziness (37.2%) and vertigo (34.1%) (Table 2). Symptom triggers related to positional changes, visual stimulation, and head movement were generally similar across the diagnoses (*p* > 0.39) (Table 2). There were also no significant differences in episodic symptom duration (*p* = 0.291) or frequency (*p* = 0.263). Patients generally reported prolonged but episodic symptoms lasting minutes to days, except in PPPD, where continuous symptoms had a relatively high rate (Table 2).

Inability to stand was comparable across the diagnoses (*p* = 0.624), with a notably higher frequency in Meniere's, at an overall rate of 91.3% (Table 2). Autonomic symptoms also showed similar frequency across the diagnoses (*p* = 0.225). Headache was reported at a comparable frequency (*p* = 0.11), with a range including a daily rate of 16.2% in VN to 28.7% in VM (Table 2). Photo‐ and Phonophobia were not significantly different across the diagnoses, with a range of 78.4% in VN to 92% in VM for photophobia (*p* = 0.239) and a range of 78.4% in VN to 90.8% in VM for phonophobia (*p* = 0.774) (Table 2). Reports of difficulty with daily tasks or personal care were comparable among the diagnoses (*p* = 0.974), with 95%–97% indicating at least occasional struggles. The DHI scores were comparable and categorized as moderately handicapped among the diagnoses (*p* = 0.652). The VSS scores were comparable and categorized as “severe” among the diagnoses (*p* = 0.199) (Table 2).

### 3.3 Journey From Symptoms to Diagnosis and Treatment

**FIGURE 2:**
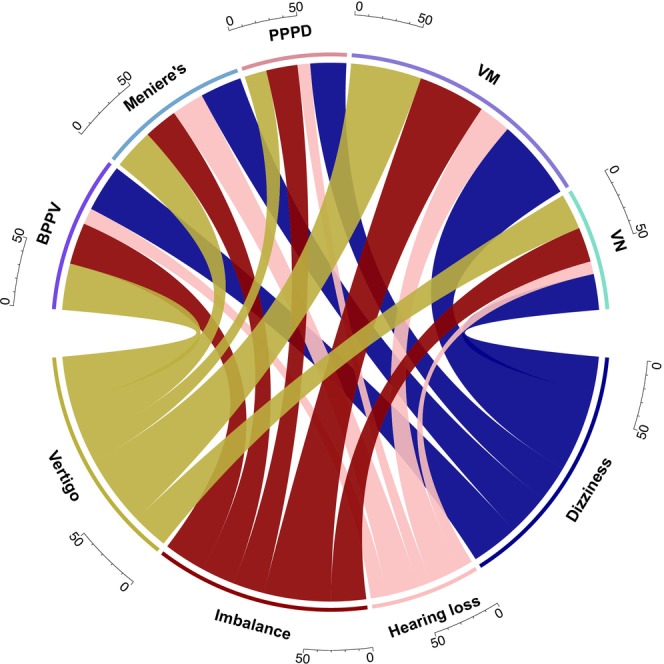
Associations among initial symptoms and five most common vestibular diagnoses. The scales represent the frequency of symptoms or diagnoses.

Respondents sought medical care in response to a broad spectrum of presentations reported as initial symptoms. The associations between these initial symptoms and the five most common vestibular diagnoses are illustrated in Figure 2 and detailed in Table S3. Dizziness as an initial symptom was not statistically different across the diagnoses and showed rates of 100% in PPPD, 90% in Meniere's, and 89.9% in VM (*p* = 0.143) (Table S3). Imbalance and unsteadiness were comparable across the diagnoses, with the rates of 85.7% in VN, 84.4% in PPPD, and 74.7% in VM (*p* = 0.376). There was no statistical difference in the rate of nausea as an initial symptom with PPPD at 68.8%, Meniere's at 65%, VM at 59.5%, BPPV at 52.9%, and VN at 51.4% (*p* = 0.483). Motion sickness was at a rate of 46.8% in VM, 37.5% in Meniere's, 37.5% in PPPD, 33.3% in BPPV, and 31.4% VN, with no statistical difference across the diagnoses (*p* = 0.457). Meniere's disease subjects had a higher rate of tinnitus (72.5%) and hearing loss (70%) compared to other diagnoses. While hearing loss prevalence was significantly higher (*p* < 0.001) than in the other common diagnoses, tinnitus did not show a statistically significant higher prevalence (*p* = 0.066). Fatigue (*p* = 0.651) and anxiety (*p* = 0.791) were also commonly reported as initial symptoms, without statistical difference across the diagnoses (Table S3).

**FIGURE 3:**
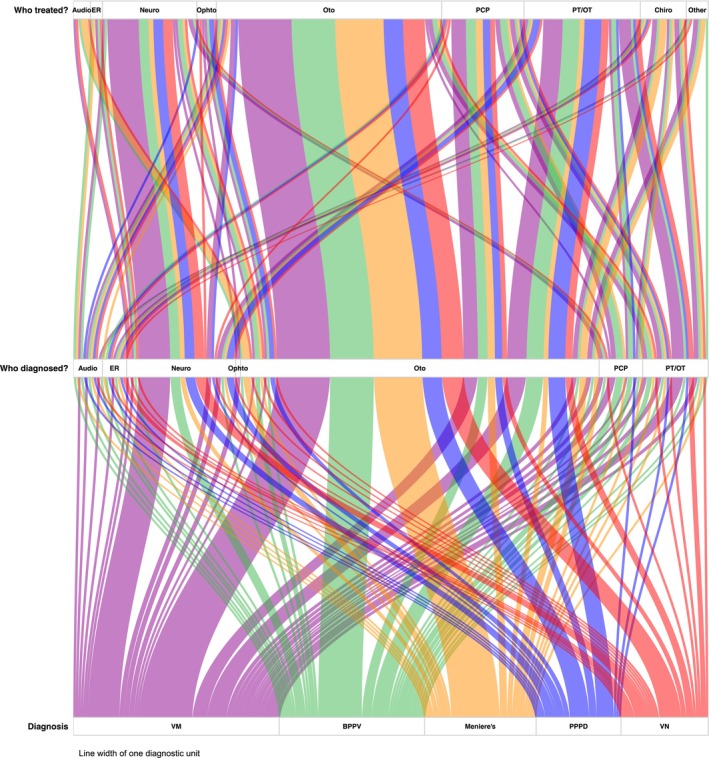
Provider Care pathways from vestibular diagnosis to treatment illustrate associations between diagnoses, diagnosing providers, and treating providers. The colors correspond to the diagnoses shown at the bottom row. Audio, audiologist; BPPV, benign paroxysmal positional vertigo; Chiro, chiropractor; ER, emergency room provider; Neuro, neurologist; Ophto, ophthalmologist; Oto, otolaryngologist; PCP, primary care provider; PPPD, persistent postural perceptual dizziness; PT/OT, physical therapist/occupational therapist; VM, vestibular migraine; VN, vestibular neuritis.

Figure 3 outlines the diagnosis‐to‐treatment pathway by the type of provider involved, with details in Tables S4 and S5. On average, patients reported seeing 18 providers for VM (SD = 12), 16 for BPPV (SD = 12), 18 for Meniere's (SD = 13), 18 for PPPD (SD = 11), and 18 for VN (SD = 12). Provider type did not differ significantly for either the diagnoses (*p* = 0.397) or associated treatments (*p* = 0.308) (Table 3). Notably, otolaryngologists had diagnosis to treatment rates of 73.9%–50.0% for Meniere's, 56.5%–33.9% for BPPV, 55.6%–27.8% for PPPD, 48.3%–32.2% for VM, and 45.9%–29.7% for VN. VM patients were diagnosed and treated by otolaryngologists at the rates of 48.3% and 32.3%, followed by neurologists at 21.8% and 20.7%, and vestibular physical therapists at 12.6% and 18.4%. Similar to VM, Meniere's, PPPD, and VN had high rates of diagnosis and treatment by otolaryngologists, followed by neurologists and vestibular physical therapists. BPPV was also mainly diagnosed and treated by otolaryngologists at 56.5% and 33.9%, followed by primary care providers at 11.3% and 14.5%, vestibular physical therapists at 9.7% and 19.4%, and neurologists at 9.7% and 12.9% (Tables S4 and S5).

**TABLE 3:** Diagnosis and treatment characteristics among common vestibular diagnoses.

Variables	VM	BPPV	Meniere's disease	PPPD	VN	*p* ^a^
Medication						0.059
No	16.3	16.1	8.7	22.9	21.6	
Currently receiving	40.7	38.7	54.3^b^	42.9	16.2^b^	
Once or more in the past	43.0	45.2	37.0	34.3	62.2	
Medication‐outcome						0.396
No change	40.8	36.0	16.7^c^	44.4	51.7^c^	
Reduced frequency	2.8	2.0	2.4	0	3.4	
Reduced severity	31.0	34.0	33.3	33.3	27.6	
Reduced frequency and severity	23.9	26.0	42.9	22.2	13.8	
Symptoms completely gone	1.4	2.0	4.8	0	3.4	
Dietary therapy						< 0.001*
No	26.7^d^	37.1	10.9^e^	37.1	56.8^d^, ^e^	
Currently receiving	36.0	25.8^f^	56.5^f^	22.9	24.3	
Once or more in the past	37.2	37.1	32.6	40.0	18.9	
Dietary therapy‐outcome						0.644
No change	40.0	28.9	26.2	45.8	27.8	
Reduced frequency	7.7	15.8	11.9	8.3	0	
Reduced severity	16.9	21.1	14.3	16.7	27.8	
Reduced frequency and severity	33.8	31.6	47.6	25.0	38.9	
Symptoms completely gone	1.5	2.6	0	4.2	5.6	
Vestibular rehabilitation						0.080
No	12.6	12.9	26.1^g^	0^g^	8.1	
Currently receiving	11.5	9.7	6.5	11.1	13.5	
Once or more in the past	75.9	77.4	67.4	88.9	78.4	
Vestibular rehabilitation‐outcome						0.702
No change	37.5	28.8	36.4	40.0	20.0	
Reduced frequency	4.2	5.8	3.0	5.7	5.7	
Reduced severity	15.3	25.0	18.2	20.0	37.1	
Reduced frequency and severity	41.7	40.4	42.4	31.4	34.3	
Symptoms completely gone	1.4	0	0	2.9	2.9	
Repositioning maneuver						0.005*
No	26.5^h^	3.3^h^	22.2	17.6	19.4	
Currently receiving	1.2	9.8	2.2	0	2.8	
Once or more in the past	72.3	86.9	75.6	82.4	77.8	
Repositioning maneuver‐outcome						0.583
No change	57.8	39.7	52.9	59.3	60.0	
Reduced frequency	6.3	6.9	2.9	3.7	8.0	
Reduced severity	7.8	17.2	23.5	7.4	8.0	
Reduced frequency and severity	17.2	25.9	14.7	14.8	20.0	
Symptoms completely gone	10.9	10.3	5.9	14.8	4.0	
Counseling						0.282
No	40.2	56.5	42.2	38.9	62.2	
Currently receiving	13.8	6.5	13.3	11.1	5.4	
Once or more in the past	46.0	37.1	44.4	50.0	32.4	
Counseling‐outcome						0.430
No change	75.5	82.7	75.0	66.7	76.9	
Reduced frequency	0	4.3	0	9.5	0	
Reduced severity	8.2	8.7	4.2	14.3	7.7	
Reduced frequency and severity	14.3	4.3	20.8	4.8	7.7	
Symptoms completely gone	2.0	0	0	4.8	7.7	
Visual therapy						0.085
No	57.3	55.0	72.7	47.1	59.5	
Currently receiving	0	1.7	0	0	5.4	
Once or more in the past	42.7	43.3	27.3	52.9	35.1	
Visual therapy‐outcome						0.861
No change	46.9	44.4	50.0	44.4	23.1	
Reduced frequency	0	3.7	0	0	0	
Reduced severity	0	0	0	0	0	
Reduced frequency and severity	18.8	22.2	28.6	16.7	30.8	
Symptoms completely gone	34.4	29.6	21.4	38.9	46.2	
Who diagnosed?						0.397
Primary care provider	8.0	11.3	4.3	2.8	2.7	
Physical or occupational therapist	12.6	9.7	6.5	5.6	13.5	
Otolaryngologist or neurotologist	48.3	56.5	73.9	55.6	45.9	
Ophthalmologist or neuro‐ophthalmologist	2.3	0	0	0	0	
Neurologist	21.8	9.7	6.5	22.2	24.3	
Emergency room physician	1.1	3.2	4.3	5.6	8.1	
Chiropractor	0	0	0	0	0	
Audiologist	3.4	6.5	4.3	5.6	2.7	
Other	2.3	3.2	0	2.8	2.7	
Who treated?						0.308
Primary care physician/Primary care nurse practitioner or Physician's assistant	11.5	14.5	10.9	13.9	13.5	
Physical or occupational therapist	18.4	19.4	6.5	33.3	16.2	
Otolaryngologist or neurotologist	32.2	33.9	50.0	27.8	29.7	
Ophthalmologist or neuro‐ophthalmologist	2.3	1.6	0	8.3	5.4	
Neurologist	20.7	12.9	6.5	11.1	16.2	
Emergency room physician	1.1	3.2	2.2	0	2.7	
Chiropractor	6.9	6.5	13.0	5.6	5.4	
Audiologist	1.1	1.6	6.5	0	5.4	
Other	5.7	6.5	4.3	0	5.4	
Diagnostic tests						0.869
VNG	16.5	22.0	19.6	16.7	19.4	
vHIT	11.8	8.5	10.9	11.1	5.6	
VEMP	9.4	5.1	4.3	11.1	16.7	
Imaging	23.5	20.3	8.7	22.2	13.9	
Caloric test	10.6	11.9	15.2	8.3	13.9	
Blood tests	11.8	6.8	13.0	13.9	8.3	
Auditory tests	16.5	25.4	28.3	16.7	22.2	

Abbreviations: BPPV, benign paroxysmal positional vertigo; PPPD, persistent postural perceptual dizziness; VEMP, vestibular evoked myogenic potential; vHIT, Video Head Impulse Test; VM, vestibular migraine; VN, vestibular neuritis; VNG, videonystagmography. All values are presented as percentages.

^a^ *p*‐value calculated with the Chi‐squared test.

^b^ Bonferroni‐corrected *p*‐value = 0.008.

^c^ Bonferroni‐corrected *p*‐value = 0.04.

^d^ Bonferroni‐corrected *p*‐value = 0.03.

^e^ Bonferroni‐corrected *p*‐value = 0.0002.

^f^ Bonferroni‐corrected *p*‐value = 0.024.

^g^ Bonferroni‐corrected *p*‐value = 0.027.

^h^ Bonferroni‐corrected *p*‐value = 0.005.

* *p*‐value < 0.05.

Current medication use varied across the diagnoses (Table 3), showing a trend toward statistical difference (*p* = 0.059). Meniere's patients reported the highest rate at 54.3%, significantly higher than VN at 16.2% (*p* = 0.008). Past medication use ranged from 34.3% to 62.2% and was comparable across the diagnoses (*p* = 0.396). Dietary therapy differed significantly (*p* < 0.001), and Meniere's had the highest rate (89.1%), followed by VM (73.3%). In contrast, VN had the lowest rate of dietary therapy (43.2%). Vestibular rehabilitation was most reported in PPPD (100%) with no statistical difference across the diagnoses (*p* = 0.08). Repositioning maneuver was most reported in BPPV, with 96.7% receiving at least once, showing a significant difference across the diagnoses (*p* = 0.005). Counseling was comparable (*p* = 0.282) with a range of 37.8% in VN to 61.1% in PPPD. Visual therapy was most reported in PPPD (52.9%), followed by BPPV (45.0%) and VM (42.7%), though not statistically significant across the diagnoses (*p* = 0.085). There were no significant differences in the distribution of diagnostic tests (*p* = 0.869). The most reported diagnostic tests were videonystagmography (VNG), audiometry, and imaging (Table 3). Meniere's had the lowest rate of imaging (8.7%), and VN had the highest rate of vestibular evoked myogenic potential (VEMP) (16.7%).

### 3.4 Impact on Quality of Life, Diagnosis, and Treatment

Barriers to access medical care were broadly comparable (Table 1), with no significant difference across the diagnoses (*p* = 0.269). About 30% of patients with PPPD, VM, and BPPV reported not knowing enough about their condition to effectively advocate for themselves or which type of provider to see or find. About 20% of patients with PPPD and VN believed symptoms would resolve on their own, which was reported as a barrier to seeking appropriate medical care. Factors such as wait times to see a provider, health insurance status, and the inability to take time off work were not commonly reported as significant barriers to care across the diagnoses (Table 1). Inability to work was comparable (*p* = 0.890), with Meniere's at 20%, followed by BPPV at 17.5%, VM at 16.9%, PPPD at 13.6%, and VN at 4.3% (Table 1).

The rate of effective treatments as reported by patients was repositioning maneuvers at 60.3% for BPPV, medications at 83.3% and dietary therapy at 73.8% for Meniere's, and vestibular rehabilitation at 80% for VN (Table 3). There were comparable improvements in severity and frequency of symptoms with medication use across the diagnoses (*p* = 0.396). VN patients mostly reported no improvement in symptoms with medication (51.7%), while Meniere's patients had the lowest rate of no response at 16.7% (*p* = 0.04). Dietary therapy showed comparable outcomes across the diagnoses (*p* = 0.644), with an improvement rate of 47.6% for both severity and frequency of symptoms in Meniere's, followed by 33.8% in VM (Table 3).

Vestibular rehabilitation was comparable in reducing severity and frequency of symptoms across the diagnoses (*p* = 0.702), with improvement rates of 41.7% in VM, followed by 40.4% in BPPV, 42.4% in Meniere's, 31.4% in PPPD, and 34.3% in VN (Table 3). Similarly, the effect of repositioning maneuvers was comparable across the diagnoses (*p* = 0.583), with a trend toward a higher improvement in both symptom frequency and severity for BPPV patients at 25.9%. A low rate of improvement was reported with counseling at the average rate of 25% across the diagnoses (*p* = 0.43). Visual therapy showed improvement at the average rate of 58% with a trend toward significance (*p* = 0.085).

### 3.5 Predictors of Vestibular Diagnoses

**FIGURE 4:**
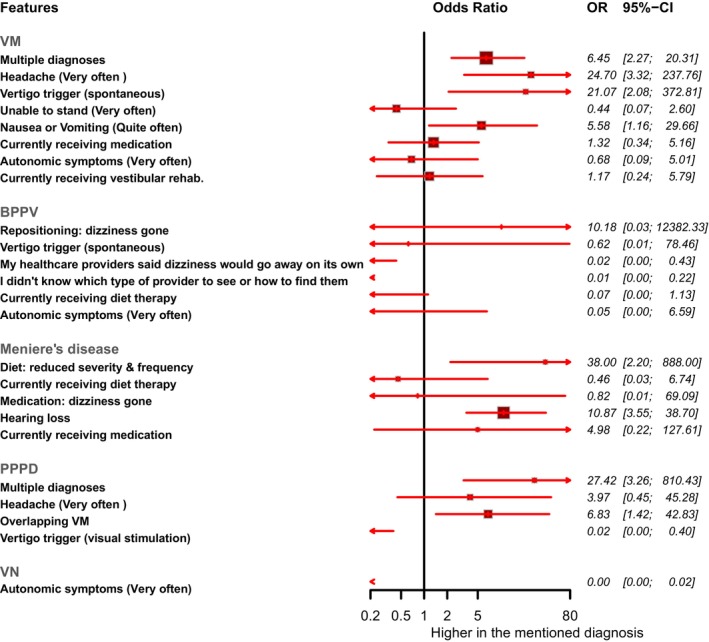
Multivariate binary logistic regression models showing predictors of the five most common vestibular diagnoses. The OR for each diagnosis is adjusted for a list of variables yielded from the recursive feature elimination. More details are provided in Table S6. Autonomic symptoms included: Hot or cold spells, fainting/feeling of blackout, heart pounding or flutter. BPPV, benign paroxysmal positional vertigo; CI, confidence interval; inf, infinity; OR, odds ratio; PPPD, persistent postural‐perceptual dizziness; VM, vestibular migraine; VN, vestibular neuritis.

We used multivariate binary logistic regression models to identify predictors for each of the five common vestibular diagnoses (Figure 4 and Tables S6 and S7). The features included in each model are listed in Table S6. Figure 4 and Table S7 include the results of the logistic regression models for these diagnoses. For VM, the significant predictors included multiple diagnosis (OR 6.45, 95% CI: 2.27–20.31, *p* < 0.001), frequent headaches (OR 24.70, 95% CI: 3.32–237.76, *p* = 0.003), spontaneous vertigo (OR 21.07, 95% CI: 2.08–372.81, *p* = 0.02), and frequent nausea/vomiting (OR 5.58, 95% CI: 1.16–29.66, *p* = 0.036). BPPV diagnosis was less likely among patients who reported being told by their provider that symptoms would go away on their own (OR 0.02, 95% CI 0.00–0.43, *p* = 0.026), not knowing which provider to see (OR 0.01, 95% CI 0.00–0.22, *p* = 0.016), or not seeking care in a timely manner (OR 0.002, 95% CI 0.00–0.43, *p* = 0.045). For Meniere's, a significant predictor was the improvement in symptoms with dietary therapy (OR 38.00, 95% CI 2.20–888.0, *p* = 0.016). Significant predictors for PPPD were multiple diagnoses (OR 27.42, 95% CI 3.26–810.43, *p* = 0.011), vertigo induced by visual stimulation (OR 0.02, 95% CI 0.00–0.40, *p* = 0.016), and overlap with VM diagnosis (OR 6.83, 95% CI 1.42–42.83, *p* = 0.03). For VN diagnosis, a significant predictor was lower frequency of autonomic symptoms (OR 0.00006, 95% CI: 0.00–0.02, *p* = 0.005).

## 4 Discussion

The current study draws on cross‐sectional data collected from the VeDA registry. The dataset includes detailed self‐reported information on the quality, timing, duration, and triggers of symptoms, as well as validated vestibular symptom scales. It also captures diagnosis, treatment types, provider specialties, and self‐reported treatment outcomes. In addition, the registry collects psychosocial factors including barriers to care, health insurance coverage, and employment status. Key strengths of this dataset include patterns of comorbidity, diagnostic overlap, and the influence of provider specialty on diagnosis, treatment, and potential gaps in care for patients with vestibular symptoms. Also, predictors of the most common vestibular diagnoses were identified using multivariate models.

The patient cohort was predominantly composed of middle‐aged, white, non‐Hispanic women—a demographic trend that aligns with previous reports suggesting a higher prevalence of vestibular disorders in women [21, 22]. Black, Hispanic, and Asian individuals with vestibular disorders were underrepresented. Healthcare access barriers among patients with vestibular disorders are likely contributing to this observed racial disparity [13, 23]. While VeDA has supported patients with vestibular disorders for over three decades and has recently made efforts to reach more diverse communities [24], challenges remain, particularly for those with limited access to digital tools and online resources.

The female predominance in vestibular disorders is well recognized, along with an age‐related rise in prevalence [21, 25, 26, 27]. Strikingly, patients reported seeing an average of 15 providers for their symptoms, underscoring the diagnostic complexity and fragmented care often associated with vestibular conditions. This may also reflect participation of patients with more persistent and complex vestibular presentations in this registry, while also capturing the common, real‐world experience of many vestibular patients. The high rate of multiple diagnoses (66.3%) and an average of about two diagnoses per patient further emphasize the clinical overlap and uncertainty surrounding these disorders [28]. The number of providers involved did not differ across diagnoses, indicating that, for example, patients with BPPV who are suitable for faster diagnosis and treatment required care from as many providers as those with more complex conditions, such as PPPD. Interestingly, BPPV diagnosis was more likely in patients who did not report being told their symptoms would resolve on their own, knew which provider to see, or sought timely medical care. Despite the high number of provider encounters across diagnoses, many reported infrequent visits, with over 16% seen less than once a year, and over 7.5% receiving no continuous care at all. This suggests gaps in access or continuity of care and is consistent with previous reports of higher annual healthcare costs for vestibular patients [7, 10]. Additionally, the high rates of inability to work (18.9%) and retirement (35.4%) among the mostly younger cohort in this study underscore the serious burden of vestibular symptoms on daily functions, as shown in previous studies [8, 10, 26, 29, 30, 31, 32].

The clinical landscape of vestibular disorders in our cohort reveals both diagnostic overlap and shared symptoms across conditions. VM was the most common diagnosis, followed by BPPV and Meniere's disease, with many patients carrying multiple diagnoses—most notably VM co‐occurring with BPPV, PPPD, and Meniere's disease. This overlap has been recognized previously and reflects the complexity and uncertainty many patients face in their journey, especially in cases involving VM, where symptoms often intersect with other vestibular conditions [33, 34, 35, 36, 37, 38, 39, 40]. Despite its high prevalence, VM currently lacks a dedicated ICD‐10 code, limiting a large‐scale analysis of associated clinical trends in these patients. VM represents a recognized link between migraine and vestibular dysfunction [33, 40, 41, 42]. However, not all patients who present with both vestibular and migraine symptoms necessarily meet criteria for VM. The high rate of diagnostic overlap reported by patients indicates gaps in making this distinction and points to challenges in achieving accurate diagnoses [40]. This is particularly important because VM is primarily diagnosed based on clinical symptoms with no definitive biomarker [33, 40, 41]. In some cases, vestibular disorders may trigger migraine symptoms rather than being caused by migraine itself. Although these overlapping symptoms may appear similar, they can reflect different underlying mechanisms. Recognizing these differences is critical for making accurate diagnosis and providing effective treatment. When vestibular symptoms from another disorder occur alongside migraine, a secondary migraine syndrome must be considered rather than primary VM [41]. A previous study found this distinction may vary across different clinical disciplines; for instance, only 4.3% of neurotologists suspected VM in patients presenting with headache and vestibular symptoms, compared with 82% of neurologists [43].

Despite different diagnoses, patients reported largely similar symptom experiences, with no significant differences in how they described vestibular symptoms, imbalance, or associated triggers. Notably, lightheadedness, described as a sensation of faintness, was frequently reported as a vestibular symptom by patients. This underscores the complexity of how patients convey their symptoms, highlighting the challenge of distinguishing vestibular disorders based on symptom profiles alone [4]. The close physiological link between the vestibular and autonomic systems may also explain such overlap in patient‐reported symptoms [44, 45]. Across all patient groups, the functional impact of symptoms was consistently high, reflected in widespread reports of difficulty with daily activities and significant levels of disability [8, 9].

Dizziness, imbalance, nausea, motion sensitivity, and fatigue were common as initial symptoms across all diagnoses, reinforcing the challenge of distinguishing between vestibular disorders based on presenting symptoms alone. Meniere's disease stood out with higher rates of tinnitus and significantly more hearing loss, supporting its distinct audiological profile. There was also high overlap in reported use of treatments across diagnoses, with only dietary therapy reported more frequently in Meniere's disease. Current medication use was also higher in Meniere's disease compared to VN. Overall, these trends highlight a lack of diagnosis‐specific management strategies, likely influenced by both the overlapping nature of symptoms and overlapping vestibular diagnoses. Together, these factors may contribute to the variability and inconsistency observed in treatment patterns.

About half of patients across all diagnoses consulted otolaryngologists, though treatment rates were relatively lower. Despite the involvement of multiple providers, no clear patterns emerged in who diagnosed or treated different vestibular conditions. This likely reflects the inherent complexity of symptoms and the challenges clinicians face in establishing accurate diagnoses and effective pathways for treatment. Although there was no distinct pattern of provider involvement in our cohort, a multidisciplinary assessment and timely referrals can reduce fragmented care, shorten time to diagnosis, and ultimately improve patient outcomes [46, 47, 48]. Some respondents reported receiving a diagnosis from PTs or OTs, though it's unclear whether these were vestibular‐trained specialists. While PTs are trained to independently evaluate, diagnose, and treat patients within their scope of practice, they do not provide a medical diagnosis [49]. Given the patient‐reported nature of the data, some participants may have misinterpreted functional evaluations as formal medical diagnoses.

Studies suggest that up to 45% of patients with vestibular symptoms present to PCPs [7, 50]. However, our findings show that only a small proportion of diagnoses or treatments were attributed to PCPs. This gap highlights the need for enhanced training among PCPs to appropriately triage, diagnose, treat, or refer patients with vestibular symptoms. Improving diagnostic skills is particularly important in conditions like BPPV that can be treated promptly once identified. This gap also extends beyond primary care to other key specialties that frequently evaluate patients with vestibular symptoms, including otolaryngology, vestibular physical therapy, and neurology. Despite the high prevalence and disabling impact of vestibular disorders, trainees in these specialties often receive limited or no formal exposure to vestibular physiology, clinical examination techniques, and diagnostic reasoning in their training curricula [51, 52]. In modern‐day practice, training in vestibular and ocular motor disorders is especially relevant because the underlying functions are well‐characterized, anatomically distinct, and consist of clearly defined subclasses that can be reliably stimulated, measured, and interpreted. In this context, vestibular ocular functions can serve as sensitive clinical markers, aiding in diagnosis, assessing disease severity, and monitoring rehabilitation progress. The arrival of new video‐oculography platforms at the bedside and advancements in clinical examination technology have made this kind of hands‐on training both feasible and essential [53, 54, 55].

With regard to predictors of vestibular diagnoses, the findings from the regression models support known clinical associations and validate the accuracy of patient‐reported experiences, reinforcing their reliability as a source of clinical insight. This further strengthens confidence in the validity of patient‐reported data and suggests that the diagnostic complexity and overlap observed in the dataset are more likely a consequence of clinical care pathways and the assignment of multiple diagnoses, rather than inaccuracies in reporting. VM was, as expected, strongly associated with symptoms linked to migraine pathophysiology—frequent headaches, spontaneous vertigo, and recurrent nausea or vomiting. While the overall distribution of headache did not differ significantly across diagnoses, it was a significant predictor only in VM patients—highlighting its diagnostic relevance in this group. Notably, having multiple diagnoses also emerged as a significant predictor of VM, again reflecting the diagnostic complexity and symptom overlap often seen in these patients. In Meniere's disease, improvement with dietary therapy was a significant predictor, reinforcing its role in clinical management and diagnosis. Interestingly, patients who received reassurance that their symptoms would resolve on their own, delayed care, or were uncertain about which provider to consult were significantly less likely to report a BPPV diagnosis—highlighting how both provider communication and patient challenges in navigating health care may influence outcomes [11]. The PPPD model revealed a distinct profile, with visual‐induced vertigo as key factors along with diagnostic overlap with VM—again underscoring the multifaceted nature of this condition and its close relationship with migraine pathophysiology. Finally, VN diagnosis was linked to fewer autonomic symptoms, which aligns with its characteristic acute, isolated vestibular presentation. Overall, despite such key associations, neither providers nor treatment approaches had clear patterns for specific diagnoses, highlighting the fragmented nature of current care pathways.

While patient‐reported data offer valuable insights into patient experiences and help highlight systemic gaps in the diagnosis and management of vestibular disorders, they may also introduce certain limitations. These include potential clinical inaccuracies, recall bias, and selection or participation bias. The lack of clear, distinctive patterns in comparisons of timing, triggers, and symptom characteristics across diagnoses may partly reflect these biases, as well as the high rate of overlapping diagnoses among patients.

Another limitation is the relatively small sample size and the absence of central vestibular dysfunction from brainstem or cerebellar pathology as an important diagnostic category. This category was not presented in our analysis because it was not among the commonly reported diagnoses in this cohort; however, it is critical to identify and should not be overlooked in clinical practice. Future iterations of the registry aim to enroll larger cohorts, broaden the range of captured diagnoses, and improve data accuracy by integrating patient‐reported information with electronic medical records.

Since the survey did not capture specific medication types, our interpretation of treatment patterns does not include the therapeutic effects of individual medications. Additionally, the reported improvement with dietary therapy in Meniere's disease should be interpreted with caution, as the results show a large confidence interval and may be impacted by reporting bias rather than a true treatment effect.

Despite these limitations, our current findings align with trends observed in prior studies of patients with vestibular disorders and confirm expected diagnosis‐specific predictors, reinforcing that these patient‐reported experiences are valid and clinically meaningful.

In conclusion, vestibular symptoms impose a high burden of disability and diminish the quality of life. This cross‐sectional analysis of the VeDA registry reveals a complex clinical landscape of vestibular disorders marked by substantial demographic imbalances, diagnosis overlap, and barriers to timely, effective care. Over half of the respondents reported multiple diagnoses, with an average of nearly two diagnoses per individual patient. This substantial overlap highlights the complexity of vestibular symptoms and the difficulty of achieving diagnostic precision, particularly for vestibular migraine, which was both the most common and most frequently overlapping diagnosis. Symptom profiles were highly similar across diagnoses, with dizziness, vertigo, imbalance, nausea, and fatigue reported widely, though Meniere's disease stood out for higher tinnitus and hearing loss rates. Treatment approaches and provider involvement showed no consistent alignment with specific diagnoses, highlighting the fragmented nature of care pathways. Data analysis identified key predictors for each diagnosis, such as migraine‐related features for VM and the benefit of dietary therapy in Meniere's disease, while also confirming substantial cross‐diagnostic overlap. Collectively, these findings underscore the need for enhanced provider training and effective multidisciplinary collaboration to improve outcomes for patients with vestibular disorders.

## Author Contributions

Conception and design of the study: A.R., S.I.N., H.G.R., C.A.R., and A.K.; acquisition and analysis of data: C.A.R., P.L.G., A.R., S.I.N., H.G.R., and A.K.; drafting a significant portion of the manuscript or figures: A.R., S.I.N., A.K., H.G.R., S.A.N., M.C.S., M.T.T., F.A.G., P.L.G., and J.A.G.

## Funding

Amir Kheradmand is supported by the National Institutes of Health Grant No (K02AG083134).

## Conflicts of Interest

H.G.R., A.K., and P.L.G. are board members of VeDA. CR is the executive director of VeDA. The other authors declare no conflicts of interest.

## Supporting information


**Table S1:** Demographics, employment status, and frequency of medical visits from all respondents.
**Table S2:** Overlapping diagnoses.
**Table S3:** Initial symptoms across the top five diagnoses (reported percentages).
**Table S4:** Frequency of top five diagnoses based on the type of healthcare provider that made the diagnosis.
**Table S5:** Frequency of top five diagnoses based on the type of healthcare who treated the patients.
**Table S6:** Results of recursive feature elimination for each diagnosis.
**Table S7:** Multivariate binary logistic regression models for predictors of five most common vestibular diagnoses.

## Data Availability

Data are available upon reasonable request from VeDA.
