# Supplementary material for: Vestibular Patient Journey: Insights From Vestibular Disorders Association (VeDA) Registry
Source: Ann Clin Transl Neurol. 2026 Feb 18;13(8):1602–18. doi: 10.1002/acn3.70334 (PMC13395020; doi:10.1002/acn3.70334)
Supplement: Supplementary file 1 [file ACN3-13-1602-s001.docx]

**Supplement**

Table of Contents

[Table S1- Demographics, employment status and frequency of medical visits from all respondents.	2](#_Toc207877375)

[Table S2- Overlapping diagnoses	4](#_Toc207877376)

[Table S3- Initial symptoms across the top five diagnoses (reported percentages).	5](#_Toc207877377)

[Table S4- Frequency of top five diagnoses based on the type of healthcare provider that made the diagnosis.	6](#_Toc207877378)

[Table S5- Frequency of top five diagnoses based on the type of healthcare who treated the patients.	7](#_Toc207877379)

[Table S6- Results of recursive feature elimination for each diagnosis.	8](#_Toc207877380)

[Table S7- Multivariate binary logistic regression models for predictors of five most common vestibular diagnoses.	9](#_Toc207877381)

# Table S1- Demographics, employment status and frequency of medical visits from all respondents.

| **Demographics (127 respondents)** | **n (%)** |
| --- | --- |
| **Race** |  |
| White | 107 (84.3%) |
| Black | 0 (0.0%) |
| Asian | 2 (1.6%) |
| Other | 18 (14.1%) |
| **Ethnicity** |  |
| Hispanic | 7 (5.5%) |
| Non-Hispanic | 120 (94.5%) |
| **Employment Status (127)** |  |
| Unable to work (disabled) | 24 (18.9%) |
| Retired | 45 (35.4%) |
| Self-employed | 18 (14.2%) |
| Employed for wages (part-time or full-time) | 34 (26.8%) |
| Student | 3 (2.4%) |
| Out of work for one year or more | 3 (2.4%) |
| **Frequency of medical visits related to vestibular diagnosis (n=186)** |  |
| 1 - 3 times per month | 16 (8.6%) |
| 1 or more times per week | 6 (3.2%) |
| 2 - 6 times a year | 73 (39.2%) |
| 7 - 11 times a year | 17 (9.1%) |
| Once a year | 30 (16.1%) |
| Less than once a year | 30 (16.1%) |
| Does not see a doctor or healthcare provider for this condition or unsure | 14 (7.6%) |
| **Diagnostic Groups (n=172)** |  |
| Multiple Diagnoses | 115 (66.9%) |
| Vestibular Migraine | 87 (50.6%) |
| Benign Paroxysmal Positional Vertigo (BPPV) | 62 (36%) |
| Meniere's Disease (Endolymphatic Hydrops) | 46 (26.9%) |
| Persistent Postural Perceptual Dizziness (PPPD, formerly called Chronic subjective dizziness) | 36 (20.9%) |
| Vestibular Neuritis | 37 (21.6%) |
| Bilateral Vestibular Hypofunction | 21 (12.2%) |
| Mal de Debarquement (MdDS) | 10 (5.8%) |
| Cervicogenic dizziness | 11 (6.4%) |
| Traumatic Brain Injury (TBI)/Concussion | 8 (4.7%) |
| Orthostatic Hypotension/Postural orthostatic tachycardia syndrome (POTS) | 5 (2.9%) |
| Superior Canal Dehiscence (SCD)/Third Window Syndrome | 4 (2.3%) |
| Perilymph fistula | 4 (2.3%) |
| Acoustic Neuroma/Vestibular Schwannoma | 4 (2.3%) |
| Auto-Immune Inner Ear Disorder (AIED) | 3 (1.7%) |
| Otosclerosis | 3 (1.7%) |
| Unilateral vestibulopathy | 2 (1.2%) |
| Central vestibulopathy | 2 (1.2%) |
| Ototoxicity | 2 (1.2%) |
| Cerebellar Ataxia with Neuropathy And Vestibular Areflexia Syndrome (CANVAS) | 1 (0.6%) |
| Cardiac arrhythmia | 1 (0.6%) |
| **Healthcare Provider who Diagnosed Vestibular Condition (n=172)** |  |
| Audiologist | 6 (3.5%) |
| Emergency room physician | 5 (2.9%) |
| Neurologist | 29 (16.9%) |
| Ophthalmologist or Neuro-ophthalmologist | 3 (1.7%) |
| Otolaryngologist or Neurotologist | 90 (52.3%) |
| Physical or Occupational Therapist | 21 (12.2%) |
| Primary Care Provider | 10 (5.8%) |
| Other | 8 (4.7%) |

# Table S2- Overlapping diagnoses

| **Category** | **VM (n=87)** | **BPPV (n=62)** | **Meniere’s (n=46)** | **PPPD (n=36)** |
| --- | --- | --- | --- | --- |
| VM only | 27 (31.03%) | 0 (0.00%) | 0 (0.00%) | 0 (0.00%) |
| PPPD only | 0 (0.00%) | 0 (0.00%) | 0 (0.00%) | 4 (11.11%) |
| Meniere's only | 0 (0.00%) | 0 (0.00%) | 18 (39.110%) | 0 (0.00%) |
| BPPV only | 0 (0.00%) | 14 (22.60%) | 0 (0.00%) | 0 (0.00%) |
| VM & PPPD | 11 (12.64%) | 0 (0.00%) | 0 (0.00%) | 11 (30.55%) |
| VM & Meniere's | 8 (9.20%) | 0 (0.00%) | 8 (17.40%) | 0 (0.00%) |
| VM & BPPV | 17 (19.54%) | 17 (27.40%) | 0 (0.00%) | 0 (0.00%) |
| PPPD & Meniere's | 0 (0.00%) | 0 (0.00%) | 0 (0.00%) | 0 (0.00%) |
| PPPD & BPPV | 0 (0.00%) | 5 (8.10%) | 0 (0.00%) | 5 (13.90%) |
| Meniere's & BPPV | 0 (0.00%) | 4 (6.40%) | 4 (8.70%) | 0 (0.00%) |
| VM & PPPD & Meniere's | 2 (2.30%) | 0 (0.00%) | 2 (4.35%) | 2 (5.55%) |
| VM & PPPD & BPPV | 8 (9.20%) | 8 (12.90%) | 0 (0.00%) | 8 (22.22%) |
| VM & Meniere's & BPPV | 8 (9.20%) | 8 (12.90%) | 8 (17.40%) | 0 (0.00%) |
| PPPD & Meniere's & BPPV | 0 (0.00%) | 0 (0.00%) | 0 (0.00%) | 0 (0.00%) |
| VM & PPPD & Meniere's & BPPV | 6 (6.89%) | 6 (9.70%) | 6 (13.05%) | 6 (16.67%) |

# Table S3- Initial symptoms across the top five diagnoses (reported percentages).

| **Variables** | **VM** | **BPPV** | **Meniere’s disease** | **PPPD** | **VN** | **P-value ^a^** |
| --- | --- | --- | --- | --- | --- | --- |
| Tinnitus | 58.2 | 58.8 | 72.5 | 50.0 | 40.0 | 0.066 |
| Dizziness | 89.9 | 82.4 | 90.0 | 100.0 | 91.4 | 0.143 |
| Pain | 25.3 | 9.8 | 12.5 | 15.6 | 22.9 | 0.156 |
| Nausea | 59.5 | 52.9 | 65.0 | 68.8 | 51.4 | 0.483 |
| Imbalance | 74.7 | 70.6 | 72.5 | 84.4 | 85.7 | 0.376 |
| Fatigue | 46.8 | 43.1 | 40.0 | 56.3 | 51.4 | 0.651 |
| Vision impairment | 31.6 | 29.4 | 15.0 | 37.5 | 28.6 | 0.265 |
| Motion sickness | 46.8 | 33.3 | 37.5 | 37.5 | 31.4 | 0.457 |
| Hearing loss | 35.4 | 27.5 | 70.0 | 34.4 | 31.4 | <0.001^*^ |
| Vertigo | 79.7 | 78.4 | 90.0 | 59.4 | 88.6 | 0.014^*^ |
| Derealization/depersonalization | 24.1 | 17.6 | 20.0 | 28.1 | 11.4 | 0.441 |
| Anxiety | 45.6 | 35.3 | 40.0 | 43.8 | 37.1 | 0.791 |
| No symptom but had abnormal lab test | 10.1 | 9.8 | 10.0 | 15.6 | 8.6 | 0.898 |
| Weakness | 17.7 | 13.7 | 12.5 | 21.9 | 17.1 | 0.823 |

^a^ P-value for the Chi^2^ test.

# Table S4- Frequency of top five diagnoses based on the type of healthcare provider that made the diagnosis.

|  | Primary Care Physician | Primary Care Nurse Practitioner or Physician's Assistant | Physical or Occupational Therapist | Otolaryngologist or Neurotology | Ophthalmologist or neuro-ophthalmologist | Neurologist | Emergency Room Physician | Audiologist | Other |
| --- | --- | --- | --- | --- | --- | --- | --- | --- | --- |
| VM, N=87 (n/N %) | 5 (5.7%) | 2 (2.3%) | 11 (12.6%) | 42 (48.3%) | 2 (2.3%) | 19 (21.8%) | 1 (1.1%) | 3 (3.4%) | 2 (2.3%) |
| BPPV, N=62 (n/N %) | 5 (8.1%) | 2 (3.2%) | 6 (9.7%) | 35 (56.5%) | 0 (0.0%) | 6 (9.7%) | 2 (3.2%) | 4 (6.5%) | 2 (3.2%) |
| Meniere’s, N=46 (n/N %) | 1 (2.2%) | 1 (2.2%) | 3 (6.5%) | 34 (73.9%) | 0 | 3 (6.5%) | 2 (4.3%) | 2 (4.3%) | 0 |
| PPPD, N=36 (n/N %) | 0 | 1 (2.8%) | 2 (5.6%) | 20 (55.6%) | 0 | 8 (22.2%) | 2 (5.6%) | 2 (5.6%) | 1 (2.8%) |
| VN, N=37 (n/N %) | 1 (2.7%) | 0 | 5 (13.5%) | 17 (45.9%) | 0 | 9 (24.3%) | 3 (8.1%) | 1 (2.7%) | 1 (2.7%) |

# Table S5- Frequency of top five diagnoses based on the type of healthcare who treated the patients.

|  | Primary Care Physician | Primary Care Nurse Practitioner or Physician's Assistant | Physical or Occupational Therapist | Otolaryngologist or Neurotology | Ophthalmologist or neuro-ophthalmologist | Neurologist | Emergency Room Physician | Chiropractor | Audiologist | Other |
| --- | --- | --- | --- | --- | --- | --- | --- | --- | --- | --- |
| VM, N=87 (n/N %) | 9 (10.3%) | 1 (1.1%) | 16 (18.4%) | 28 (32.2%) | 2 (2.3%) | 18 (20.7%) | 1 (1.1%) | 6 (6.9%) | 1 (1.1%) | 5 (5.7%) |
| BPPV, N=62 (n/N %) | 8 (12.9%) | 1 (1.6%) | 12 (19.4%) | 21 (33.9%) | 1 (1.6%) | 8 (12.9%) | 2 (3.2%) | 4 (6.5%) | 1 (1.6%) | 4 (6.5%) |
| Meniere’s, N=46 (n/N %) | 4 (8.7%) | 1 (2.2%) | 3 (6.5%) | 23 (50.0%) | 0 | 3 (6.5%) | 1 (2.2%) | 6 (13.0%) | 3 (6.5%) | 2 (4.3%) |
| PPPD, N=36 (n/N %) | 5 (13.9%) | 0 | 12 (33.3%) | 10 (27.8%) | 3 (8.3%) | 4 (11.1%) | 0 | 2 (5.6%) | 0 | 0 |
| VN, N=37 (n/N %) | 3 (8.1%) | 2 (5.4%) | 6 (16.2%) | 11 (29.7%) | 2 (5.4%) | 6 (16.2%) | 1 (2.7%) | 2 (5.4%) | 2 (5.4%) | 2 (5.4%) |

# Table S6- Results of recursive feature elimination for each diagnosis.

| **Diagnosis** | **Variables selected via RFE** |
| --- | --- |
| **VM** | 1. Multiple diagnoses 2. Headache 3. Vertigo triggers 4. Inability to stand 5. Nausea/vomiting frequency 6. Medication use 7. Autonomic symptoms 8. Vestibular rehabilitation result |
| **BPPV** | 1. Repositioning maneuver outcome 2. Multiple diagnoses 3. Repositioning maneuver 4. Vertigo triggers 5. Symptom duration 6. Barriers to care 7. Imbalance quality 8. Dietary therapy 9. Autonomic symptoms |
| **Meniere’s disease** | 1. Dietary therapy outcome 2. Dietary therapy 3. Medication effect 4. Hearing loss 5. Medication use |
| **PPPD** | 1. Multiple diagnoses 2. Headache 3. Barriers to care 4. Counseling outcome 5. VM diagnosis 6. Vertigo triggers |
| **VN** | 1. Vertigo triggers 2. Barriers to care 3. Autonomic symptoms 4. Dizziness description 5. Symptom duration 6. Diagnostic tests |

BPPV, benign paroxysmal positional vertigo; PPPD, persistent postural perceptual dizziness; RFE, recursive feature elimination; VM, vestibular migraine; VN, Vestibular Neuritis.

**Table S7- Multivariate binary logistic regression models for predictors of five most common vestibular diagnoses.**

| **Variables** | **Multivariate ^a^**  **OR (95%CI)** | **P-value^*^** |
| --- | --- | --- |
| **VM** | | |
| Multiple diagnosis (yes) | 6.45 (2.27– 20.31) | <0.001 |
| Headache (ref: no headache) |  |  |
| Very often (most days) | 24.70 (3.32– 237.76) | 0.003 |
| Vertigo triggers (ref: no vertigo) |  |  |
| It occurs spontaneously | 21.07 (2.08- 372.81) | 0.02 |
| Nausea or Vomiting (ref: no) |  |  |
| Quite often (every week) | 5.58 (1.16–29.66) | 0.036 |
| **BPPV** | | |
| Barriers (ref: no barrier) |  |  |
| My healthcare providers told me that my dizziness would go away on its own | 0.02 (0.00–0.43) | 0.026 |
| I didn't know which type of provider to see or how to find them | 0.01 (0.00–0.22) | 0.016 |
| I did not seek care in a timely manner | 0.002 (0.00-0.43) | 0.045 |
| **Meniere’s disease** | | |
| Dietary therapy (ref: not receiving therapy) |  |  |
| Reduced the severity and frequency of the symptoms | 38.00 (2.19– 888.00) | 0.016 |
| **PPPD** | | |
| Multiple diagnosis (yes) | 27.42 (3.26– 810.43) | 0.011 |
| Vertigo triggers (ref: no vertigo) |  |  |
| It is induced by visual stimulation | 0.02 (0.00– 0.40) | 0.016 |
| Overlapping VM diagnosis | 6.83 (1.42–42.83) | 0.03 |
| **VN** | | |
| Autonomic symptoms ^b^ (ref: no) |  |  |
| Very often (most days) | 0.00006 (0.00–0.02) | 0.005 |

BPPV, benign paroxysmal positional vertigo; CI, confidence interval; inf, infinity; OR, odds ratio; PPPD, persistent postural perceptual dizziness; VM, vestibular migraine; VN, Vestibular Neuritis.

^*^ Only variables with a P-value<0.05 are reported in the table.

^a^ The Odds ratio for each diagnosis is adjusted for variables as listed in Table S6.

^b^ Autonomic nervous symptoms included: hot or cold spells, fainting/feeling of blackout, heart pounding or flutter.
